# Supplementary material for: Novel methodology for assessing total recovery time in response to unexpected perturbations while walking
Source: PLoS One. 2020 Jun 3;15(6):e0233510. doi: 10.1371/journal.pone.0233510 (PMC7269230; doi:10.1371/journal.pone.0233510)
Supplement: S2 Table — (DOCX) [file pone.0233510.s003.docx]

| **Table S2. Median and range of recovery times, for Level 12, initial contact and toe off perturbations, by group, condition, and perturbation group type.** | | | | | | | | | | | | | | | | | | | | | | | | | |
| --- | --- | --- | --- | --- | --- | --- | --- | --- | --- | --- | --- | --- | --- | --- | --- | --- | --- | --- | --- | --- | --- | --- | --- | --- | --- |
|  | | Medio-Lateral Perturbations | | | | | | | | | | | | | | | | Anterior-Posterior Perturbations | | | | | | | |
|  |  | PLtIcLt | | PLtIcRt | | PLtToLt | | PLToRt | | PRtIcLt | | PRtIcRt | | PRtToLt | | PRtToRt | | TmIcLt | | TmIcRt | | TmToLt | | TmToRt | |
|  |  | SL | SW | SL | SW | SL | SW | SL | SW | SL | SW | SL | SW | SL | SW | SL | SW | SL | SW | SL | SW | SL | SW | SL | SW |
| OA ST | Median  (n) | 5.88  (5) | 4.96  (5) | 5.52  (3) | 5.73  (3) | 4.26  (3) | 9.00  (3) | 5.78  (6) | 5.29  (6) | 7.43  (3) | 15.11  (3) | 9.15  (6) | 11.92  (6) | 5.33  (5) | 5.60  (5) | 10.86  (2) | 5.53  (2) | 4.90  (1) | []  (0) | []  (0) | []  (0) | 4.72  (4) | 6.33  (3) | 5.25  (8) | 5.92  (7) |
|  | Min | 5.22 | 3.94 | 4.72 | 5.52 | 3.69 | 7.15 | 4.61 | 4.53 | 6.33 | 4.57 | 6.33 | 4.57 | 4.51 | 4.50 | 6.39 | 4.67 | 4.90 | [] | [] | [] | 3.82 | 5.74 | 3.76 | 2.02 |
|  | Max | 9.89 | 11.84 | 13.21 | 9.51 | 5.53 | 12.02 | 10.53 | 11.49 | 10.87 | 17.08 | 12.71 | 17.08 | 14.44 | 14.68 | 15.32 | 6.39 | 4.90 | [] | [] | [] | 10.57 | 11.57 | 11.17 | 12.79 |
| OA DT | Median  (n) | 4.92  (5) | 6.41  (5) | []  (0) | []  (0) | 3.94  (2) | 5.14  (2) | 8.08  (7) | 5.51  (7) | 4.42  (3) | 6.51  (3) | 4.97  (7) | 5.94  (7) | 4.89  (7) | 4.89  (7) | 5.80  (5) | 10.13  (5) | 9.86  (2) | 5.57  (1) | 9.78  (1) | 7.02  (1) | 5.38  (3) | 8.72  (3) | 4.80  (7) | 7.36  (7) |
|  | Min | 4.18 | 4.63 | [] | [] | 3.37 | 5.11 | 4.50 | 4.36 | 3.68 | 4.42 | 3.62 | 4.36 | 4.58 | 4.10 | 3.50 | 4.50 | 8.27 | 5.57 | 9.78 | 7.02 | 2.34 | 5.17 | 3.46 | 5.75 |
|  | Max | 9.26 | 12.01 | [] | [] | 4.51 | 5.17 | 17.72 | 9.03 | 9.06 | 8.84 | 9.06 | 8.84 | 7.07 | 5.48 | 11.86 | 18.48 | 11.45 | 5.57 | 9.78 | 7.02 | 6.16 | 20.18 | 17.94 | 24.12 |
| YA ST | Median  (n) | 5.33  (1) | 5.91  (1) | []  (0) | []  (0) | 5.64  (1) | 5.06  (1) | 6.58  (6) | 5.53  (6) | []  (0) | []  (0) | 4.66  (2) | 7.49  (2) | 5.13  (6) | 5.74  (6) | 4.48  (4) | 10.22  (4) | 3.46  (2) | 22.82  (1) | 5.59  (1) | 5.59  (1) | []  (0) | []  (0) | 18.74  (1) | []  (0) |
|  | Min | 5.33 | 5.91 | [] | [] | 5.64 | 5.06 | 4.27 | 3.68 | [] | [] | 3.90 | 5.42 | 4.22 | 4.83 | 4.03 | 4.26 | 3.06 | 22.82 | 5.59 | 5.59 | [] | [] | 18.74 | [] |
|  | Max | 5.33 | 5.91 | [] | [] | 5.64 | 5.06 | 6.87 | 7.50 | [] | [] | 5.42 | 9.56 | 10.52 | 6.85 | 5.16 | 21.63 | 3.87 | 22.82 | 5.59 | 5.59 | [] | [] | 18.74 | [] |
| YA DT | Median  (n) | 5.52  (1) | 14.55  (1) | 4.65  (1) | 4.65  (1) | 4.71  (4) | 4.45  (4) | 4.97  (5) | 5.53  (5) | 4.30  (3) | 6.50  (3) | 6.40  (4) | 6.81  (4) | 9.70  (6) | 5.21  (5) | 4.64  (7) | 5.75  (7) | 9.75  (2) | 5.99  (3) | 3.63  (2) | 19.20  (1) | 4.32  (1) | 8.08  (1) | 4.89  (1) | 10.31  (1) |
|  | Min | 5.52 | 14.55 | 4.65 | 4.65 | 4.41 | 4.41 | 4.63 | 4.32 | 4.20 | 4.70 | 4.20 | 4.70 | 6.73 | 4.57 | 4.18 | 4.18 | 4.52 | 2.40 | 3.54 | 19.20 | 4.32 | 8.08 | 4.892 | 10.31 |
|  | Max | 5.52 | 14.55 | 4.65 | 4.65 | 4.99 | 11.12 | 24.27 | 7.25 | 8.51 | 11.57 | 14.64 | 11.57 | 11.95 | 7.82 | 6.35 | 17.39 | 14.98 | 22.72 | 3.72 | 19.20 | 4.32 | 8.08 | 4.892 | 10.31 |

Min = minimum; Max = maximum; OA= older adults; YA = young adults; ST = single task; DT = dual task; PLtIcLt = platform left initial contact left; PLtIcRt = platform left initial contact right; PRtIcLt = platform right initial contact left; PRtIcRt = platform right initial contact right; TmIcLt = treadmill initial contact left; TmIcRt = treadmill initial contact right; PLtToLt = platform left toe off left; PLtToRt = platform left toe off right; PRtToLt = platform right toe off left; PRtToRt = platform right toe off right; TmToLt = treadmill toe off left; TmToRt = treadmill toe off right; SL = step length; SW = step width.
